# Venoarterial extracorporeal membrane oxygenation in adults with cardiogenic shock

**DOI:** 10.3389/fcvm.2026.1851908

**Published:** 2026-06-25

**Authors:** Jamel Ortoleva, Patrick M. Wieruszewski, Amy French, Jerome C. Crowley, Eriberto Michel, William Riley, David Convissar, Dominic V. Pisano

**Affiliations:** 1 Department of Anesthesiology, Boston Medical Center, Boston, MA, United States; 2 Department of Anesthesiology, Mayo Clinic, Rochester, MN, United States; 3 Department of Pharmacy, Mayo Clinic, Rochester, MN, United States; 4 Department of Cardiovascular Medicine, Lahey Hospital & Medical Center, Burlington, MA, United States; 5 Corrigan Minehan Heart Center ICU, Massachusetts General Hospital, Boston, MA, United States; 6 Harvard Medical School, Boston, MA, United States; 7 Department of Anesthesiology, Massachusetts General Hospital, Boston, MA, United States; 8 Division of Cardiac Surgery, Department of Surgery, Massachusetts General Hospital, Harvard Medical School, Boston, MA, United States; 9 Department of Perfusion, Boston Medical Center, Boston, MA, United States; 10 Department of Critical Care, AdventHealth, Orlando, FL, United States

**Keywords:** cardiogenic shock (CS), ECMO (Extracorporeal membrane oxygenation), mechanical circulatory support (MCS), refractory shock, venoarterial (VA) ECMO

## Abstract

Cardiogenic shock in adults continues to carry a high mortality despite advances in cardiac care, interventions and critical care. A major advancement in cardiogenic shock care came with the advent of temporary mechanical circulatory support including the intra-aortic balloon pump, ventricular assist devices, and venoarterial extracorporeal membrane oxygenation (VA ECMO). VA ECMO is a type of temporary mechanical circulatory support used in selected patients with cardiogenic shock that have failed to be adequately supported by less invasive approaches including vasoactive medications, volume optimization, and other temporary mechanical circulatory support. VA ECMO is resource intensive support strategy that requires expertise and cooperation from multiple specialties. Complications during VA ECMO support are common and include bleeding, acute kidney injury, stroke, mechanical complications during cannulation, hemolysis, and limb ischemia. VA ECMO circuits drain blood from the venous system, oxygenate and remove carbon dioxide, and return it to the arterial system, providing biventricular support. VA ECMO may be deployed peripherally (through femoral or internal jugular veins and returning blood to axillary or femoral arteries), or centrally from the right atrium to the aorta. Vascular access used for peripheral VA ECMO is large and includes arterial cannulas (usually 15 to 19 French), venous cannulas (usually 21 to 25 French), and a distal perfusion catheter (5 to 8 French). VA ECMO causes significant physiologic changes including reduced pulmonary blood flow, an inflammatory response, increases in left ventricular afterload, dual circulation when initiated through most peripheral sites, and coagulopathy. Management considerations for patients supported by VA ECMO are complex for multiple reasons including the differing approaches to the underlying cause of cardiogenic shock (for example, ischemic vs. non ischemic etiologies), patient comorbidities, whether the goal is recovery, heart transplant, or dischargeable ventricular assist device. Weaning from VA ECMO is a complex process with multiple possible approaches, and decannulation is most commonly accomplished through surgical or percutaneous approaches. There is a paucity of literature on VA ECMO and most guidance is based on retrospective data and expert opinion. What follows is an overview of VA ECMO for cardiogenic shock.

## Introduction

With advances in critical care, coronary intervention, temporary mechanical circulatory support (tMCS), durable ventricular assist devices as well as the ever-widening scope of heart transplantation, the management of cardiogenic shock has evolved in recent years. Despite this, mortality during initial hospitalization remains high at approximately 50% (1). Many definitions exist for cardiogenic shock, but broadly speaking, cardiogenic shock is the result of insufficient oxygen delivery to tissue and organs due to primary cardiac compromise (2). Cardiogenic shock may be due to ischemic and non-ischemic etiologies, with differences in management depending on diagnosis. Initial management of cardiogenic shock includes a combination of hemodynamic stabilization with vasoactive agents and tMCS if needed, volume optimization, revascularization if indicated, supportive care for end-organ dysfunction, and consideration of advanced therapies in some cases. Use of tMCS may be as a bridge to recovery, a bridge to durable therapy, or a bridge to transplant.

The introduction of tMCS has significantly changed cardiogenic shock management, with proponents pointing out that patients in previously terminal states may now be potentially recoverable with the use of tMCS. The first widely used tMCS device is the intra-aortic balloon pump (IABP). While IABP use is common in cardiogenic shock, studies have not found a survival advantage with its use as primary support (3, 4). A recent trial on the use of transvalvular microaxial flow pumps (tvMFP) found a survival advantage in patients with cardiogenic shock due to myocardial infarction (5). Of note, in the as-treated group of the aforementioned tvMFP trial, there was no survival difference, and complications rates in the tvMFP group were higher which brings into question the robustness of the result (5). A large retrospective review from a German healthcare database of 4,088 patients found that tvMFP may be associated with improved survival in patients with myocardial infarction related shock compared to VA ECMO, but selection bias could not be controlled for given the retrospective nature (6). However, while tMCS has increasingly been used to support patients with cardiogenic shock, recent negative clinical trial results, including those showing harm without benefit, highlight the need for further research (Table 1) (7–12).

**Table 1:** Randomized trials of VA ECMO for cardiogenic shock (7–10).

Study	Years	Patient population	Results
Extracorporeal Life Support in Cardiogenic Shock Complicating Acute Myocardial Infarction Brunner et al.	2015–2019	42 (21 VA ECMO, 21 control) Myocardial infarction related cardiogenic shock	Primary outcome: no difference in LVEF at day 30,∼50% in both. Secondary outcomes: no difference in mortality (19% VA ECMO versus 33% no VA ECMO, *p* = 0.37). Longer mechanical ventilation (*p* = 0.03) and ICU length of stay (*p* = 0.001) for the VA ECMO group.
Venoarterial extracorporeal membrane oxygenation or standard care in patients with cardiogenic shock complicating acute myocardial infarction: the multicentre, randomised EURO SHOCK trial Banning et al.	2020–2022	35 (17 VA ECMO, 18 control) Myocardial infarction related cardiogenic shock Trial halted due to the COVID-19 pandemic. Initial goal: 428 patients.	5 patients randomized to VA ECMO did not receive it, 3 access complications, 2 refusal/withdrawal. Mortality at 30 days (primary outcome): 43.8% VA ECMO, 61.1% control (no statistically significant difference). Underpowered.
Extracorporeal Membrane Oxygenation in the Therapy of Cardiogenic Shock: Results of the ECMO-CS Randomized Clinical Trial Ostadal et al.	2014–2022	122 (5 excluded due to no consent, 117 analyzed) 58 immediate VA ECMO, 59 no immediate VA ECMO *73/117 (62.4%) myocardial infarction (STEMI or NSTEMI)	23/59 (39%) no immediate VA ECMO patients received it. No difference in survival, cardiac arrest, or other complications in the immediate versus delayed VA ECMO groups. Survival: 50% vs 47.5% immediate vs delayed VA ECMO.
Extracorporeal Life Support in Infarct-Related Cardiogenic Shock Thiele et al.	2019–2022	420 (3 were not randomized, 417 analyzed) 276 (of 411 recorded patients), had STEMI (67.2%)	192/209 randomized to VA ECMO received it, 26 of 208 in control received VA ECMO. Mortality: 47.8% VA ECMO, 49% control. More complications in VA ECMO group: Bleeding: 23.4% vs 9.6% (RR: 2.44, 95% CI: 1.50 to 3.95), Peripheral vascular needing intervention: 11% vs 3.8% (RR: 2.86, 95% CI: 1.31 to 6.25)

95% CI, 95% confidence interval; COVID-19, Coronavirus infectious disease 2019; LVEF, Left ventricular ejection fraction; NSTEMI, non-ST Elevation Myocardial Infarction; RR, Relative risk; STEMI, ST-Elevation Myocardial Infarction; VA ECMO, Venoarterial extracorporeal membrane oxygenation.

**Figure 1:**
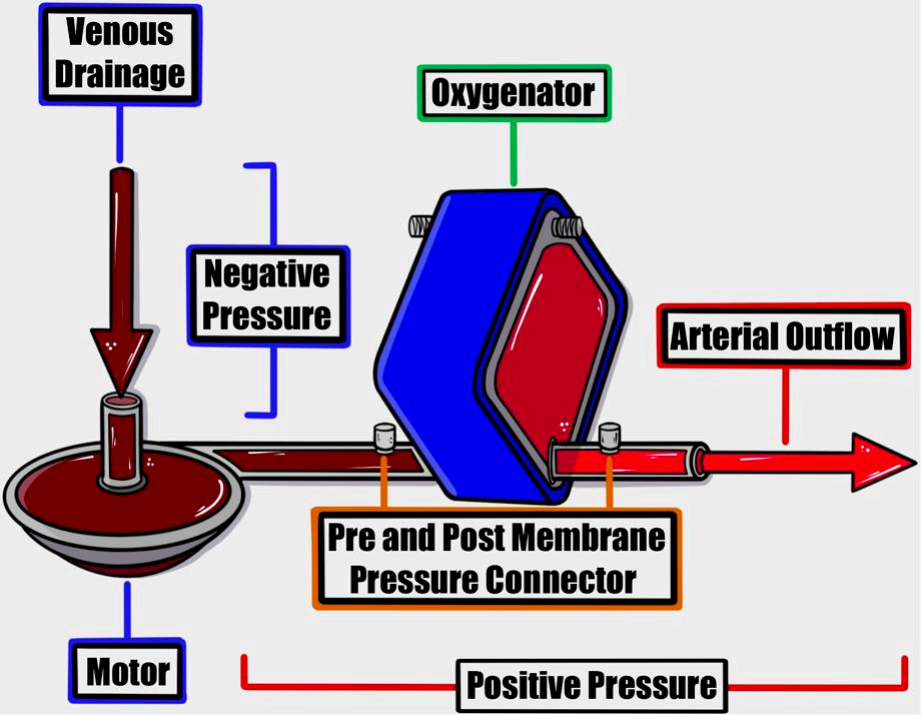
Schematic of venoarterial extracorporeal membrane oxygenation circuit. Note e dark blood (before being exposed to the oxygenator or “pre-oxygenator”) and the bright blood on the (after being exposed to the oxygenator or “post-oxygenator”).

VA ECMO is a biventricular support strategy utilized for cardiogenic shock. VA ECMO drains blood via a venous cannula to a pump, propels the blood through an oxygenator (or “membrane lung”), and re-infuses it to the arterial system (Figure 1) (13). VA ECMO provides both hemodynamic and respiratory support and may be utilized for multiple indications, including cardiogenic shock, obstructive shock (specifically, pulmonary embolism), hemodynamically unstable arrhythmias, peri-procedural hemodynamic support, and for cardiac arrest (known as extracorporeal cardiopulmonary resuscitation, or eCPR) (13–15). VA ECMO is most commonly deployed via the femoral vessels due to ease of access and rapid initiation of support in essentially any location, including outside of a medical facility (13, 14, 16). VA ECMO rapidly stabilizes hemodynamics and supports organ function while the etiology of decompensation is characterized and plan is made. VA ECMO initiation results in important physiologic changes that must be anticipated and include dual circulation for patients with most peripheral VA ECMO strategies, negative pressure in the venous system from the centrifugal pump, reduced cardiac preload, reduced arterial pulsatility, platelet dysfunction, inflammation, and acquired von Willebrand factor deficiency (13, 17–19). While VA ECMO as a support device that has had a significant impact on the management of cardiogenic shock, the morbidity and mortality in patients requiring this intervention is high and patient selection is complex. VA ECMO is among the most resource intensive support strategies and requires a multi-disciplinary team for management and treatment of complications. Research on VA ECMO is rapidly growing, but many unanswered questions remain.

The objective of this narrative review is to provide a comprehensive overview of the use of VA ECMO for cardiogenic shock with a specific focus on decision-making, circulatory physiology, cannulation and management strategies, and complications. For information regarding the use of VA ECMO for eCPR, the reader is directed to the literature (15). For information regarding tMCS decision making, including device selection, in patients with cardiogenic shock, the authors are referred to the literature on this subject, which is based on expert opinion due to the lack of randomized studies (20–22). As this was a narrative review, a structured systematic literature search was not performed. Instead, we identified relevant articles and topics from the published literature and the group's combined clinical experience.

### History of VA ECMO

The first use of VA ECMO was in 1972 for a patient with respiratory failure-induced cardiac compromise after a motor vehicle accident (23). After this report, VA ECMO was utilized in a neonate with meconium aspiration syndrome (24). As enthusiasm for VA ECMO grew, the results of a randomized trial of VA ECMO for acute respiratory failure (1979) disappointed, with survival below 10% in both arms (25). Despite these disappointing results, advancements in ECMO technology continued to occur (26). In 1989, the Extracorporeal Life Support Organization (ELSO) was founded, and is the largest international organization focused on ECMO and extracorporeal life support. A trial in patients with acute respiratory distress syndrome (ARDS) found no difference in survival between venovenous (VV) ECMO for carbon dioxide removal and routine care (27). A randomized trial of patients with severe ARDS found that referral to a center capable of VV ECMO resulted in improved outcomes (28). The use of ECMO to support respiratory failure continued to increase, particularly during the 2008–2009 H1N1 pandemic (29, 30).

Throughout this time period, the most common use of VA ECMO in the adult population (in the United States) was myocardial compromise after cardiac surgery (postcardiotomy shock) (31). Postcardiotomy shock remained the most common indication until 2011, after which cardiogenic shock for other reasons became the most common indication (31). The use of tMCS continued to increase with a notable spike after the United Network for Organ Sharing (UNOS) heart transplant allocation system changed in October of 2018; the new allocation system strongly prioritizes patients supported by tMCS, which resulted in a significant increase in tMCS use (32). The use of VA ECMO as a bridge to transplant steadily increased throughout this period with improving outcomes, likely related to increased clinician experience and improvements in critical care (33, 34). VA ECMO is also used as a means of stabilization and bridging to other devices with the goal of transplant with comparable outcomes to VA ECMO alone (35). A recent large retrospective review of 17,087 patients between 2016 and 2021 from the ELSO registry found that before 2018, there were 214 patients bridged to heart transplant, while after 2018 there were 409 patients, with a statistically significant increase in use of the technology for this indication, a statistically significant increase (36). The same investigation found that 58 patients were transitioned to left ventricular assist device (LVAD) before 2018 and 368 were transitioned to LVAD after 2018 (36).

Recent investigations of VA ECMO for cardiac arrest and myocardial infarction have yielded mixed results. One small, single center study of 30 patients (29 analyzed) randomized subjects aged 18 to 75 with ventricular fibrillation refractory to three attempts of defibrillation to eCPR vs. conventional CPR (37). Patients undergoing eCPR had significantly higher survival to hospital discharge (43% vs. 7%) and at 3 and 6 months (43% vs. 0% for both) (37). Another randomized trial of 122 patients (117 randomized) with cardiogenic shock to early VA ECMO (58 patients) or a “watch and wait” approach for hemodynamic deterioration (59 patients) with a composite primary endpoint of resuscitated cardiac arrest, death, or need for another tMCS device (9). The investigators found a non-statistically significant difference, in the composite primary endpoint (63.8% in early VA ECMO and 71.2% in the watch and wait approach) in both groups (9). Two randomized clinical trials published in 2023 yielded negative results (10, 38). One trial of 160 patients (134 analyzed) examined the use of VA ECMO for eCPR in out of hospital cardiac arrest with the finding that survival was equivalent (20% for eCPR vs. 16% for conventional CPR) (38). Of note, in this trial the median time from arrest to VA ECMO initiation was 74 min with interquartile range (IQR) of 63 to 87 min (38). Yet another randomized trial on eCPR for out of hospital cardiac arrest was stopped due to futility at an interim analysis after 256 patients were randomized with the finding that 31.5% of patients receiving eCPR were discharged with neurologically favorable status vs. 22% in patients undergoing standard resuscitation (39). Given these and other results, some have questioned current VA ECMO usage trends given the unclear benefit in randomized trials (12).

### VA ECMO mechanism of action and physiology

**Figure 2:**
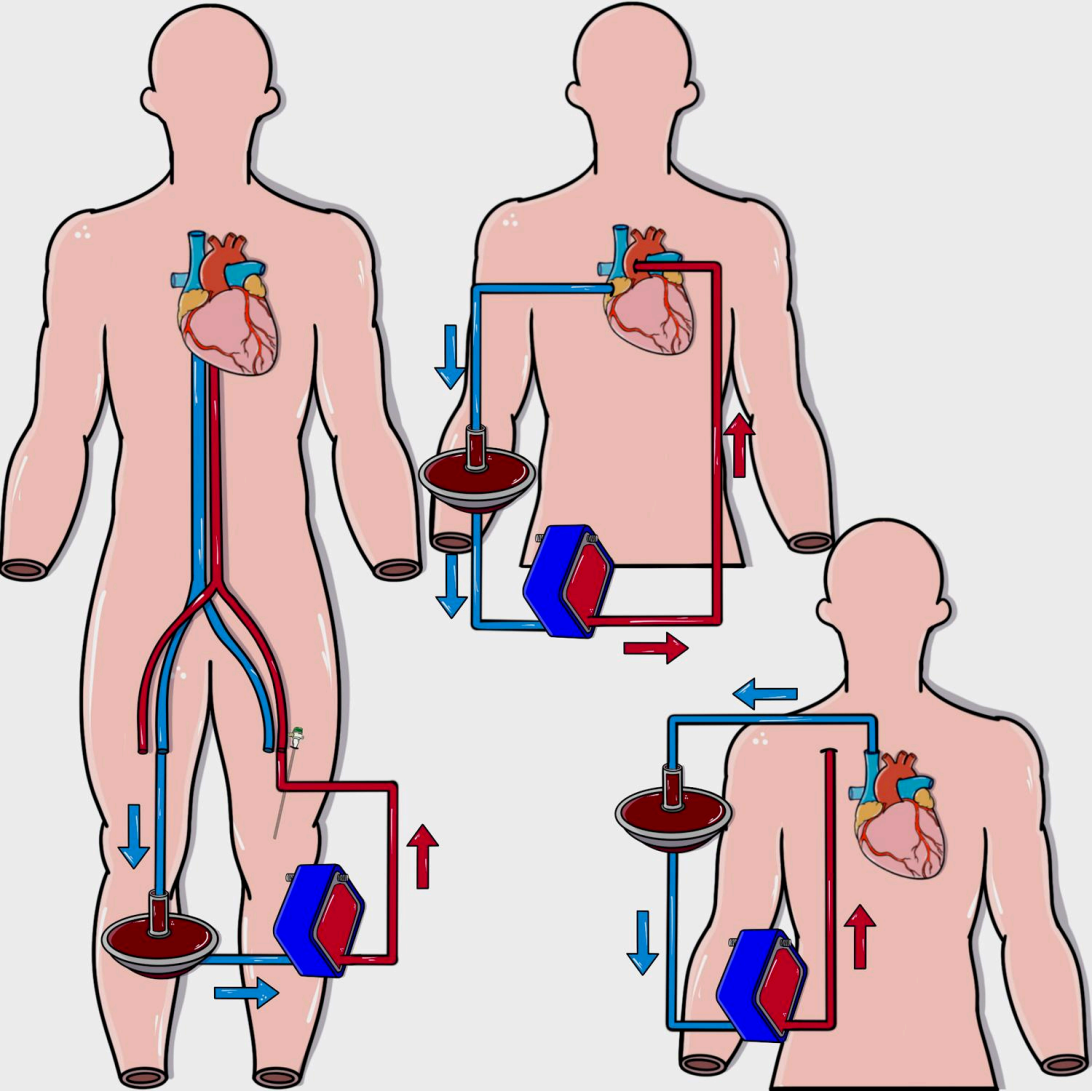
Three examples of venoarterial extracorporeal membrane oxygenation (VA ECMO). *Left:* Peripheral VA ECMO with femoral venous drainage and femoral arterial return with distal perfusion catheter in the left lower extremity superficial femoral artery. *Upper Middle:* Central VA ECMO with drainage from the right atrium and return to the ascending aorta. *Bottom Right:* Peripheral VA ECMO via Internal jugular vein drainage and return to the right axillary artery.

All configurations of VA ECMO drain blood from the venous system (via negative pressure) to a motor which propels the blood through an oxygenator, where oxygen is added and carbon dioxide is removed, and return the blood to the arterial system (Figures 1, 2, Table 2). Draining from the venous system with reinfusion to the arterial system provides hemodynamic and respiratory support. A centrifugal pump is used in essentially all contemporary adult VA ECMO circuits, which returns blood perpendicular to the direction of drainage. This contrasts to axial flow pumps which drain and return blood along a straight line. The centrifugal pump flow mechanism of ECMO circuits is relatively more hemocompatible but more sensitive to increases in afterload.

**Table 2:** Comparison of venoarterial extracorporeal membrane oxygenation cannulation configurations (31, 40–42).

Specifics	Femoral	Axillary	Central
Typical Cannula sizes	Arterial: 15–19 Fr Venous: 21–25 Fr	Arterial: 15–19 Fr Venous: 21–25 Fr	Arterial: 18–24 Fr Venous: Varies
Cannulation considerations	Percutaneous or cutdown	Percutaneous or Surgical cutdown with 8–10 mm graft	Sternotomy or thoracotomy
Nature of flow	Retrograde	Mostly antegrade	Antegrade
Impact on LV afterload	Moderate to high	Modest to moderate	Minimal
Advantages	Ease of Access, rapid deployment, does not require OR, less invasive	Ambulation, access when technically challenging (e.g., femoral vascular disease, morbid obesity)	Higher blood flow rate, LV decompression
Disadvantages	Dual circulation (differential oxygenation/carbon dioxide), limb ischemia, infection, retrograde blood flow, difficulty ambulating	Nerve injury, upper extremity hyperperfusion (with graft usage), graft bleeding, ischemia with cannula usage (less common)	Highly invasive access, bleeding, infection, restricted mobility

Fr, French size; LV, Left ventricle; mm, millimeters.

**Figure 3:**
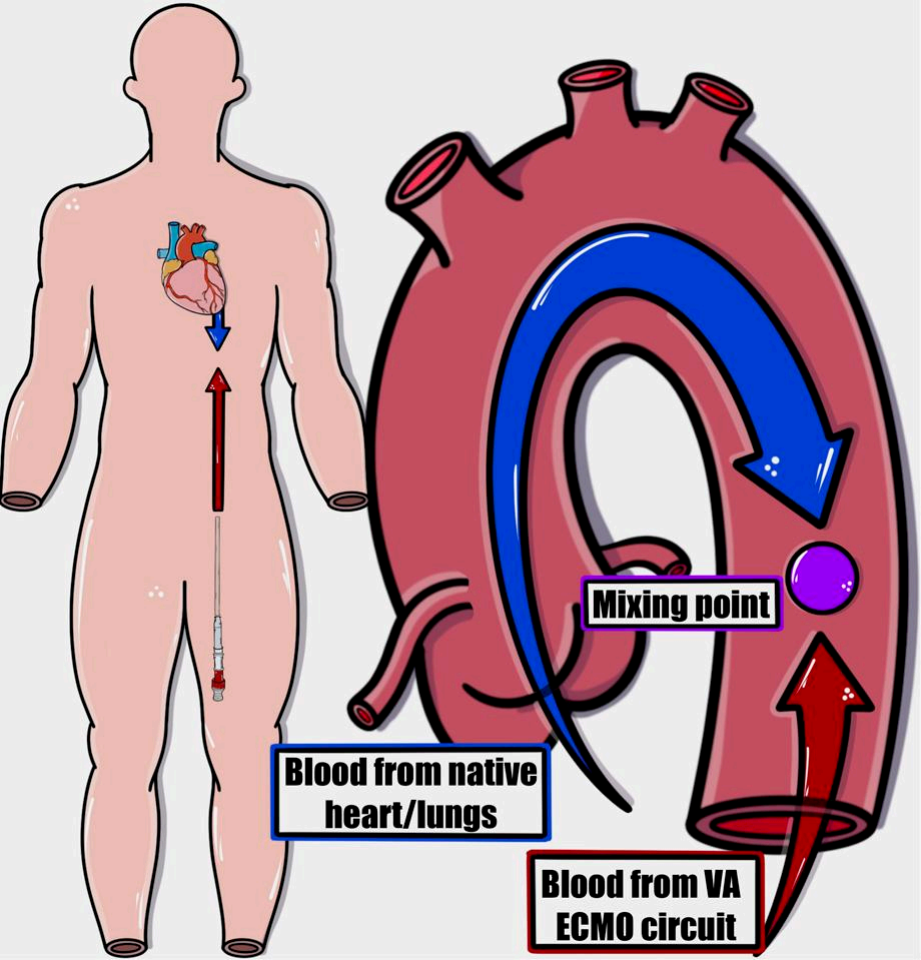
Demonstration of dual circulation in patients supported by femoral venous—femoral arterial venoarterial extracorporeal membrane oxygenation (VA ECMO). The mixing point is the location where blood from the native heart and lungs (the blue arrow) meets blood from the VA ECMO circuit (the red arrow).

VA ECMO initiation results in profound physiologic changes including significant reductions in blood flow through the pulmonary vasculature, an inflammatory response from blood contact with foreign surfaces, reduced systemic pulsatile flow, hemodilution from a rapid bolus of crystalloid within the circuit (∼700 mL), and reductions in platelets and von Willebrand factor. VA ECMO, particularly when the arterial cannula is in a femoral artery, results in retrograde flow that can increase afterload on the left ventricle due to retrograde arterial blood flow. In a patient with a failing left ventricle, this increase in afterload can reduce aortic valve opening and increase left ventricular pressures which worsen left atrial pressure and contribute to pulmonary edema or even hemorrhage (43). Interestingly, a recent publication assessing variations in circuit flow during either VA ECMO or VA ECMO with IABP found that pulmonary capillary wedge pressure (PCWP) elevations are infrequent (6%) (44). The most common form of VA ECMO, (femoral arterial and venous cannulas), results in a region of blood with high oxygen and low carbon dioxide levels distal to the “mixing point”, the point where blood flow from the native heart meets continuous blood flow from the ECMO circuit (Figures 2, 3) (18). The result of these different flows is blood with different oxygen and carbon dioxide levels depending on the location sampled. This dual circulation phenomenon can have important physiologic consequences including cerebral and myocardial hypoxemia and excess renal excretion of bicarbonate with alkalotic post-oxygenator blood (45). These physiologic changes must be understood and anticipated; occasionally, cannula repositioning or venoarterial venous ECMO may be needed for cerebral and myocardial oxygenation (13, 46).

### Indications and contraindications for VA ECMO for cardiogenic shock

The decision to proceed with VA ECMO initiation for management of cardiogenic shock is complex. Most programs utilize a multidisciplinary team that includes a combination of intensivists with cardiovascular critical care experience, heart failure cardiologists, interventionalists, and surgeons, with the aim of understanding all aspects of the clinical scenario. Firm numerical criteria for the initiation of VA ECMO in cardiogenic shock, such as lactate levels, amount of vasoactive support doses, or pH have not been established. Among the most well-known guideline documents on this subject from ELSO state that:

“VA ECMO should be considered for cardiogenic shock within 6 h of occurrence, refractory to conventional pharmacological and fluid therapy, and in patients with reversible cardiocirculatory collapse or those eligible for alternative cardiocirculatory assistance, for example, ventricular assist devices (VADs) or transplantation.” (13)

In terms of shock severity at VA ECMO initiation, a large (12,106 patient) ELSO database review found that the majority of patients with cardiogenic shock undergoing VA ECMO cannulation were Society for Cardiovascular Angiography and Intervention (SCAI) stages C or D (SCAI shock stages: A through E with E being most severe) and that increasing shock severity at the time of intervention was associated with higher hospital mortality (47). While experts will agree that VA ECMO initiation for cardiogenic shock is best timed before significant organ dysfunction or cardiac arrest occurs, vasoactive inotropic score thresholds and the duration of a trial of non-ECMO care are not known. Further complicating this issue is that there are multiple definitions of cardiogenic shock (48). Large retrospective reviews suggest that delays in VA ECMO initiation are associated with increased mortality in patients admitted with cardiogenic shock: VA ECMO initiation should not be delayed once the decision for support has been made (49).

While the only absolute contraindications to VA ECMO cannulation are patient refusal and inability to obtain vascular access (for example, severe peripheral vascular disease or dissection at the site of proposed arterial access), risk tolerance and willingness to proceed vary significantly by program. When VA ECMO is declined it is often due to combination of contraindications that in aggregate are felt to negate the benefits of the technology.

Many relative contraindications to VA ECMO exist (Table 3). For example, end stage cardiac dysfunction without a durable support option, significant aortic regurgitation, and severe peripheral vascular disease are all relative contraindications (Table 3). Additionally, end stage disease of other organs that are not amenable to transplantation, and acute multiorgan failure felt not to be recoverable are important relative contraindications. From a neurologic perspective, intracranial hemorrhage, ischemic stroke, and poor baseline neurologic function are relative contraindications to VA ECMO initiation. Other considerations include the inability to transfuse blood products (except for brief support duration), the presence of a terminal diagnosis such as metastatic malignancy with life expectancy less than one year, poor functional status, and active bleeding requiring significant transfusion (13, 50–52). Finally, the presence of inferior vena cava filters and congenital lesions such as an incomplete vena cava can complicate or make femoral VA ECMO cannulation impossible.

**Table 3:** Absolute and relative contraindications to VA ECMO (13).

**Absolute Contraindications**	**Relative Contraindications**
Patient or decision maker refusal No exit strategy (unrecoverable cardiac function without the possibility of LVAD, transplant, or procedural option to correct pathology) DNR/DNI/CMO status No vascular access options (for example, completely occluded arteries and very high venous thrombus burden)	**Neurologic:** Poor neurologic status Chronic minimally conscious state Ischemic/hemorrhagic stroke Severe traumatic brain injury **Cardiovascular:** Severe peripheral vascular disease Severe or moderate to severe aortic insufficiency Inferior vena cava filter (though some models accommodate cannulas) Aortic dissection **Pulmonary:** COPD requiring home oxygen or other interstitial lung disease without the possibility of transplant Severe pulmonary hypertension and no transplant option **Gastrointestinal:** Severe Liver Cirrhosis with no transplant option **Renal:** End stage renal disease **Hematologic/Oncologic:** Refusal of blood transfusion/difficulty finding a cross match for blood transfusion Active significant bleeding (except pulmonary artery hemorrhage because reducing blood flow through the lungs can improve bleeding) Malignancy with life expectancy under one year Significant coagulopathy **General:** Poor functional status Unrecoverable Multi organ failure Infected at proposed cannulation site

Note that there is variation from center to center and that not all contraindications taken from the referenced article. CMO, Comfort measures only; COPD, Chronic obstructive pulmonary disease; DNI, Do not intubate; DNR, Do not resuscitate; LVAD, Left ventricular assist device.

Ethical implications with VA ECMO can be extremely complex with issues including varying inclusion and exclusion criteria depending on the institution, the morbidity of the technology without clear evidence of benefit in randomized trials, financial implications and decisions regarding withdrawal and lack of care escalation. Literature on this subject is relatively sparse but growing and includes case-based discussions as well as reviews and opinion pieces (53, 54). Navigating the principles of autonomy, beneficence, nonmaleficence, and justice and complicated with VA ECMO. First, the lack of randomized data and high-quality literature showing benefit makes decision making complex. Second, given the critical pre-ECMO state, patients are often not able to make their own decisions regarding the initiation for support. Finally, given the significant financial implications of VA ECMO usage, production pressure could play a role in the use of the technology. For example, in one study of Medicare Fee-for-Service inpatient data from October 2019 to December 2022, mean cost for VA ECMO was $264,021 with a standard deviation of $157,882 (55).

Other important considerations for VA ECMO cannulation decision-making include the long-term outcome of the patient and avoidance of a “bridge to nowhere”. For example, a patient with endocarditis and cardiogenic shock that is not a candidate for procedural intervention would not benefit from VA ECMO cannulation due to the lack of an exit strategy. Another example is a patient with cardiogenic shock due to myocardial infarction that is not a candidate for revascularization or advanced heart failure therapy options.

### Risk calculators and decision making for cannulation

Multiple risk calculators exist for patients with cardiogenic shock being considered for VA ECMO cannulation. The most well-known example is the “Survival After Veno-Arterial ECMO” or SAVE score (56). This calculator was constructed from an ELSO registry cohort of 3,846 patients with cardiogenic who underwent VA ECMO cannulation from 2000 to 2013. (56) The score includes variables encompassing pre-ECMO conditions, such as cannulation indication, pulse pressure, history of cardiac arrest prior to cannulation, and mechanical ventilation duration and includes chronic renal disease as defined by a glomerular filtration rate below 60 mL/min/1.73m^2^ (56). Other scores have also been created using conventional and machine learning methods including PREDICT (which examines survival based on variables within the first 6 to 12 h of VA ECMO cannulation), IMPACT (examining mortality within 72 h of VA ECMO initiation), and ENCOURAGE (mortality at 6 months after intensive care stay for acute myocardial infarction requiring VA ECMO) (57–59). These three scores are important advancements as VA ECMO prediction tools but limited in their scope, as they are from relatively small cohorts. Other prediction scores utilizing machine learning including the RESCUE-24, ECMO PAL, and prediction models for neurologic injury in VA ECMO (60–62). These prediction models utilized machine learning and were trained on datasets of over 1,000 patients. Of note, risk calculators for a general population of critically ill patients such as the sequential organ failure assessment score (SOFA) and the addition of right ventricular function to the SOFA score (SOFA-RV) have been utilized in small studies with initially promising results, though more work is needed in this area (63, 64).

While risk calculators have their place in VA ECMO cannulation decisions, derivation from retrospective databases relies on accurate and complete documentation of comorbidities and acute pathologies. Missing data is common, and thus prediction models do not fully replicate clinician intuition or current practice and can quickly become outdated. Finally, prediction models should ideally utilize pre-cannulation datapoints as a decision aid tool for appropriateness of initiation, but multiple models utilize data after the initiation of support. While post-support data assists “post initiation” prognostication, it does not assist with decision-making for initiation. Given the heavy resource utilization of VA ECMO, improvements are necessary before prediction models can be widely applied as decision tools. At the present time, reliance on risk calculators for VA ECMO is not recommended given that many come from single center analyses and are often insufficiently validated. Ideally, prospective datasets are constructed to assist with the creation of new models with biologically plausible variables.

### VA ECMO configurations

VA ECMO may be deployed through multiple access points based on urgency, vascular considerations (including peripheral vascular disease, vessel caliber, and venous thrombosis), and indication (13, 50, 65). Rare cannulation locations in adults including the innominate or carotid arteries are infrequently described and not discussed (66, 67). The most common cannulation strategy for VA ECMO is femoral venous and femoral arterial access. Alternative access sites such as axillary artery, subclavian artery, and ascending aorta cannulation are also used (Figure 2) (50). Femoral access is always the first choice for patients undergoing eCPR with deviation only if vascular anatomy forbids femoral access. For VA ECMO for cardiogenic shock, femoral access is still the predominant modality. Femoral access safely enables over 5 L/min of blood flow depending on cannula size.

Femoral VA ECMO is also easily decannulated and does not occupy supradiaphragmatic vasculature making central venous access more straightforward. Drawbacks of femoral VA ECMO include the possibility for differential oxygenation leading to cerebral hypoxemia, limb ischemia (partly mitigated by smaller cannulas and routine distal perfusion catheter), deep venous thrombosis after decannulation, and more challenges with ambulation, though it is still possible (68). Femoral cannulation may also confer a lower risk of stroke and bleeding compared to other cannulation strategies but a higher risk of infection and limb ischemia (69).

Axillary or subclavian access for VA ECMO is often pursued in patients with significant femoral arterial peripheral vascular disease (40). Alternatively, it is deployed to facilitate mobilization when combined with right internal jugular venous access for drainage (70–72). Right axillary arterial access is often selected due to the added advantage of the elimination of cerebral hypoxemia by flowing hyperoxygenated blood before the origin of the carotid arteries. Additional advantages include patient comfort and ease of lower extremity movement. Disadvantages to this strategy include the potentially higher risk of neurologic events and bleeding compared to femoral cannulation (69). Additionally, while axillary cannulation and decannulation may be performed percutaneously, they are more likely to require operating room management.

Central VA ECMO cannulation involves placement of the arterial cannula in the ascending aorta with or without right atrial access for drainage (for example, femoral venous drainage and ascending aortic cannulation would still constitute central VA ECMO). Advantages of this strategy include the lack of differential oxygenation, lack of cannula occlusion of peripheral arteries to cause direct limb ischemia, and the ability to place larger cannulas and generate higher blood flow rates (6 L/min) than any other configuration. Additionally, arterial line sampling may be at any location due to the direct flow of hyper oxygenated blood into the ascending aorta. However, in multiple circumstances including post cardiotomy shock and primary graft dysfunction after heart transplant, meta-analyses suggest central cannulation has multiple important disadvantages compared to peripheral cannulation including the highly invasive nature (requiring sternotomy or thoracotomy for both cannulation and decannulation), bleeding, and potentially increased mortality (31, 41, 73, 74). Additionally, given the need to access the mediastinal space, central VA ECMO confers a risk of mediastinitis that is not seen with peripheral strategies.

In summary, multiple VA ECMO cannulation strategies exist and although femoral access is most common, consideration of alternative access sites is important in certain clinical scenarios. Severe peripheral vascular disease, concomitant significant pulmonary compromise, and the need for high blood flow rates are circumstances to consider alternative access. The risks and benefits of each cannulation strategy should be considered with the understanding that central VA ECMO is most morbid, is associated with the highest complication rate, and may confer increased mortality.

### Mortality and complications of patients supported by VA ECMO

**Figure 4:**
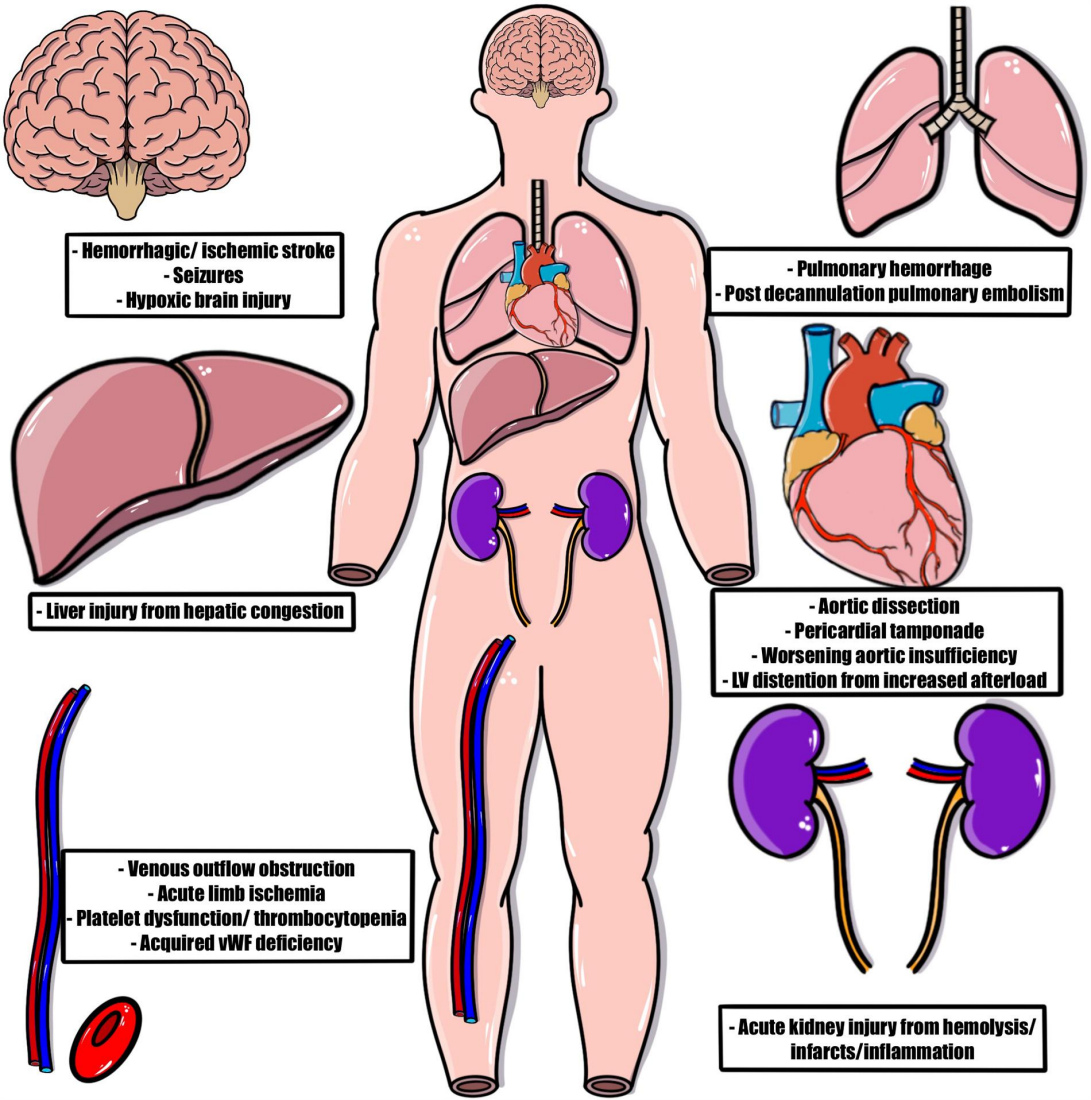
Examples of complications, by organ system, that can occur in patients supported by venoarterial extracorporeal membrane oxygenation. LV: Left ventricular, vWF: von Willebrand factor.

Due to the presence of cardiogenic shock prior to cannulation as well as the highly invasive nature of ECMO, adverse events are common (Figure 4, Table 4). For example, one analysis of a 10,207-patient VA ECMO sample from a registry in Germany found 20% (2,044) of patients experienced major bleeding complications, 7.4% (751) had limb ischemia, 7% (711) suffered abdominal ischemia, and 1% (105) suffered a stroke (90). Expressed as events per one thousand hours, the 2022 ELSO registry report noted that renal replacement therapy (1.655), cannula (0.871) and surgical (0.751) bleeding, hemolysis (0.282), gastrointestinal hemorrhage (0.271), and extremity ischemia (0.241) are the most common complications (91). In this same report, survival following VA ECMO (defined as being alive 24 h after decannulation) from the 2009 to 2022 period was 62.6% and survival at hospital discharge was 44.2% (out of a total of 45,830 adult VA ECMO patients) (91). In a report on VA ECMO after cardiac surgery from 2000 to 2020, hospital mortality was 60.3% (1,219 of 2,021 patients) and varied by duration of VA ECMO support: 66.1% (429 of 649), 50.8% (394 of 776), 58.6% (154 of 263), 72.7% (242 of 333) for 0 to 3, 4 to 7, 8 to 10, and over 10 days of support, respectively (92). In addition to mortality, patients requiring VA ECMO support frequently develop organ dysfunction.

**Table 4:** Complications occurring in venoarterial extracorporeal membrane oxygenation (VA ECMO) (6, 18, 44, 75–89).

**Organ System**	**Complications**	**Selected Incidence of Complications**
General	Mortality	55.8%–67%
Neurological	Ischemic/Stroke Hypoxic Brain injury Seizure Brain Death	Overall: ∼15%–20% Stroke (Ischemic): ∼3%–8% Stroke (Hemorrhagic): ∼1.5%–3% Seizure: ∼2% Brain Death: ∼5%
Cardiac	Tamponade Perforation Arrhythmias Root Thrombosis Pulmonary Edema LV Thrombus Vasodilation (inflammation)	Tamponade: 2.6%
Hematologic	Hemolysis, Thrombocytopenia HIT Bleeding (medical) Bleeding (cannula) Bleeding (surgical)	Hemolysis: 4%–4.8% Thrombocytopenia: 23.9% HIT: 3.5% Bleeding (medical/cannula/surgical site): 12.6%/23.5%/20.8% of hemocompatibility complications Red blood cell transfusion: 81%
Vascular and Limb	Limb Ischemia Amputation Compartment Syndrome/fasciotomy Vessel injury from cannulation Aortic Dissection	Limb Ischemia: 6.5–8.3% Limb Amputation: 0.7% Compartment Syndrome/fasciotomy: 2.7% Compartment Syndrome: 2.4% Fasciotomy: 1.7%
Renal	AKI or Renal RRT	AKI or RRT: 12.1%–53% RRT: 5.9%–44%
Physiologic	Differential Oxygenation Increased LV Afterload	6% increase in pulmonary capillary wedge pressure with increasing VA ECMO Flows
Infectious	Infection (any location)	Sepsis: 19%, Septic Shock: 8%

AKI, acute kidney injury; HIT, Heparin induced thrombocytopenia; LV, left ventricle; RRT, renal replacement therapy.

Neurologic complications in patients supported by VA ECMO are common and likely underestimated in retrospective reviews due to the high mortality rate. Contributing factors to neurologic complications include hypoperfusion from shock and embolic phenomenon (from the circuit, and from within the heart, and less commonly, paradoxical emboli). VA ECMO circuit related complications such as from large, acute changes in arterial partial pressure of carbon dioxide (P_a_CO_2_) also likely contribute to neurologic sequalae (75, 76, 93, 94). Microemboli as detected by transcranial doppler have been detected in 35 of 100 VA ECMO patients in one prospective observational study but an association with stroke was not established (95). In a well-known retrospective review of the ELSO registry examining patients supported by VA ECMO from 1992 to 2013, the incidence of neurologic complications was 682 of 4,522 patients (15.1%) and when separated by indication, the 3,005 patients supported for strictly cardiac indications had a 5.1% rate of brain death, 2.0% rate of seizures, 4% rate of infarction, and 1.9% rate of cerebral hemorrhage (77). Additionally, in this work the mortality of patients suffering from neurologic complications was 89% compared to 57% in those without neurologic complications (77). Another ELSO registry review of VA ECMO from 2013 to 2017 for non-eCPR indications found that 401 of 10,342 (3.9%) patients had ischemic stroke (with mortality 76%) while 229 of 10,342 (2.2%) patients had hemorrhagic stroke (with mortality 86%) while patients without either had a 56% mortality (78). Finally, a study of patients from the ELSO registry from 2012 to 2021 found 659 of 20,297 patients (3.2%) suffered ischemic stroke and 287 of 20,297 (1.4%) suffered hemorrhagic stroke while 38 patients (0.2%) suffered both (79). In this work, mortality of patients found to have ischemic and hemorrhagic strokes was much higher than those without (65% vs. 36% and 72% vs. 37% for ischemic and hemorrhagic strokes, respectively) (79). Taken together, the current evidence suggests neurologic complications have a significant association with mortality and methods of prevention and detection should be utilized.

Bleeding (all types) and other hematologic issues are the most common complications in patients supported by VA ECMO (91, 96). Among adults, the ELSO registry defines a bleeding complication (except intracranial) as the requirement of at least three units of blood in a 24 h period (97). Potential areas of bleeding include cannula sites, surgical sites, gastrointestinal, intracranial, airway, retroperitoneal, and oropharyngeal. Mechanisms for bleeding include cannulation-related injury, the use of anticoagulation (most commonly heparin, but occasionally other agents such as direct thrombin inhibitors), acquired von Willebrand deficiency, and thrombocytopenia (42, 80, 81, 98, 99). A retrospective review found that bleeding in VA ECMO occurs at a rate of 44.3% with a 22.3% rate of major bleeding and is associated with a nearly 9% absolute increase in the rate of death or disability as a composite outcome (96). A large registry review found that bleeding occurred in 32.8% of patients (82). One retrospective review of both VV and VA patients noted that although prior literature found higher rates of hemolysis, the authors found that only 4% of patients supported by VA ECMO had significant hemolysis as defined by a plasma free hemoglobin level above 500 mg/L (83). A large review of the ELSO registry found that in VA ECMO patients, 571 of 11,984 (4.8%) experienced hemolysis (82). Heparin induced thrombocytopenia (HIT) is a known complication with VA ECMO due to the frequent use of heparin for anticoagulation, with one single center retrospective review finding 13 of 373 (3.5%) patients with confirmed HIT and 53 of 373 (14.2%) with anti-platelet factor 4 antibodies (84). A meta-analysis on HIT in ECMO (all types) found a 4.2% incidence (85). In a review of the ELSO registry, 8,457 hemocompatibility events (for example: bleeding, thrombosis, hemolysis) were noted in 5,285 of 11,984 patients (44.1%) with bleeding at cannula sites (23.5% of events) and surgical sites (20.8% of events) being most common while circuit clots composed 18.4% of events (82). In this review, 12.6% of hemocompatibility events were “medical bleeding” defined as hemorrhagic stroke, pulmonary bleeding and gastrointestinal bleeding (82). Finally, mortality in patients suffering from hemocompatibility events was significantly higher than in patients without them: 54.6% vs. 64.4% (bleeding), 59.8% (thrombosis), 66.5% (both bleeding and thrombosis) (82).

Limb ischemia primarily affects patients supported by peripheral VA ECMO, impacting both morbidity and mortality (13, 100, 101). Limb ischemia is an important complication in the peripheral VA ECMO population, occurring at a rate of 0.241 events per 1,000 hours (91). In peripheral VA ECMO, and most commonly with femoral arterial access, the cannula can be fully occlusive of the artery, causing limb ischemia. The ELSO interim VA ECMO guidelines recommend that, for cannulas larger than 17 French, placement of a distal perfusion catheter, either in the common femoral artery directed into the superficial femoral artery (or directly in the superficial femoral artery), the tibial artery, or in the dorsalis pedis artery should be performed to reduce limb ischemia risk (13, 102). While prophylactic distal perfusion catheters are becoming widely adopted, even with their use the risk of limb ischemia is non-zero (103–105). While the ELSO guidelines recommend distal perfusion catheter placement when arterial cannulas over 17 French are used, they may not be needed with smaller cannulas, so long as the limb is closely monitored using Doppler and other modalities (13, 106). In one study of 7,070 patients with cardiogenic shock, the overall rate of limb ischemia was found to be 6% and in patients without tMCS it was noted to be 1.6% (1.2% in heart failure associated cardiogenic shock and 3% in myocardial infarction related cardiogenic shock) (107). In this same work, the risk of acute limb ischemia in patients supported by VA ECMO was noted to be 11.6% (107). Patients with cardiogenic shock and acute limb ischemia had higher mortality (54.7%) than those without limb ischemia (38%) (107). The incidence of limb ischemia varies by study. One review of the ELSO registry covering the time period of 2014 to 2018 comparing the differences between cardiogenic shock due to myocardial infarction or due to other etiologies found that of 432 of 6,646 (6.5%) patients had limb ischemia, 231 of 6,646 (3.48%) underwent fasciotomy, 125 of 6,646 (1.88%) developed compartment syndrome, and 54 of 6,646 (0.8%) required amputation (101). A trend towards more limb ischemia in the acute myocardial infarction group was noted (61 of 756 or 8.06% vs. 371 of 5,890 or 6.3%, *p* = 0.06), but compartment syndrome, fasciotomy and amputation were similar (101). Another study examining the location of cannulas in femoral-femoral VA ECMO analyzed patients from the ELSO registry undergoing unilateral (arterial and venous cannula on the ipsilateral side) or bilateral (cannulae on opposite sides) and found that among 19,093 patients (11,965 same-sided cannulation and 7,128 opposite-sided cannulation), there was no difference in limb ischemia that required intervention, but interestingly, bleeding, vessel repair, and in hospital mortality were lower with opposite-sided cannulation in a model adjusting for confounders (108). Finally, another large dataset review found that of 24,047 patients, 598 (2.5%) had compartment syndrome and 397 (1.7% and 66.4% of the total and compartment syndrome cohort, respectively) underwent fasciotomy (86). Given the ease of placement prior to arterial cannulation and the devastating sequelae of limb ischemia, routine prophylactic distal perfusion catheter placement seems reasonable.

Renal and hepatic complications in VA ECMO can occur due to hypoperfusion in the setting of cardiogenic shock or as a side effect of the support platform. In addition to hypoperfusion, other contributors to renal dysfunction amongst VA ECMO patients include hemolysis, transfusion, contrast administration, infection, and medications. Renal complications are common, with the need for renal replacement therapy occurring at a rate of 1.655 events per 1,000 hours of VA ECMO (91). A study examining a cohort of cardiogenic shock patients found that renal complications occurred in 964 of 7,950 patients (12.1%), and renal replacement therapy was needed in 5.9% (87). Similarly, liver dysfunction in the VA ECMO population is an important cause of morbidity and mortality, with one retrospective review of acute and chronic liver dysfunction finding that VA ECMO patients with acute liver dysfunction had a mortality of 66% compared to 55.8% for patients with chronic liver dysfunction (91, 109).

Serious VA ECMO circuit related complications are fortunately relatively uncommon but have catastrophic consequences. Circuit related problems captured by the ELSO registry include cannula issues (including the need to reposition or replace), air entrainment, circuit exchange, thrombosis, and cannula site bleeding (91). With VA ECMO for cardiac patients, a large review of the ELSO registry found that the rate of cannula problems, air entrainment, circuit exchange, circuit clots and thrombosis, and cannula site bleeding were 0.206, 0.051, 0.177, 0.225, and 0.751 per 1,000 hours, respectively (91). Contributors to these issues include anticoagulation, urgency of cannulation, cannulator experience, patient anatomy, meticulous attention to closure of venous access points from the air, and coagulopathy. Central venous access must be performed with great care and with VA ECMO circuit blood flow reductions prior to vessel access to prevent air entrainment and even guidewire aspiration into the circuit (110, 111). Accidental decannulation is rare but devastating and protocols must be in place for proper securement and monitoring of cannula positioning.

Cardiac complications in patients supported by VA ECMO include pulmonary edema, pulmonary hemorrhage, LV thrombus formation, tamponade, aortic root thrombus, and ventricular arrhythmias (13, 88). These complications occur when the LV fails and the aortic valve ceases to open with subsequent increases in left heart chamber pressures. Arterial and pulmonary artery catheter monitoring assist with early detection of LV failure and can prompt immediate action such as volume removal, inotrope initiation, positive end expiratory pressure (PEEP), and LV mechanical unloading (13). Aggressive management is necessary if pulse pressure decreases to 10 mmHg or less as this could be a sign of LV failure. Pulmonary artery pressure monitoring can discern between hypovolemia and LV failure as the etiology of low systemic arterial pulsatility. The standard goal for systemic arterial pulsatility is greater than 10 to 15 mmHg (13, 43). While it is widely accepted to initiate mechanical unloading in patients with arrhythmias, pulmonary edema, and complete lack of systemic arterial pulsatility despite non-invasive measures, hemodynamic thresholds are unknown and thresholds in the literature vary greatly (112). Algorithms for LV unloading vary in approach (13, 43, 112).

Differential oxygenation and differential carbon dioxide stem from the altered physiology of peripheral (non-right axillary) VA ECMO (18, 113). The mixing point, or location where blood from the VA ECMO circuit meets blood from the native heart defines the location at which blood proximal to this area is ejected from the native heart and is oxygenated and ventilated by the native lungs, while blood distal to this point is oxygenated and ventilated by the oxygenator (18). If the native lungs are receiving insufficient oxygen or ventilation support, or pathology such as pneumonia, pulmonary edema, pneumothorax, pulmonary embolism, ARDS, or hemorrhage exists, then the blood ejected by the native heart may be hypoxemic, hypercarbic, or both. As a result, organs and tissue proximal to the mixing point will be perfused by this hypoxemic, hypercapnic blood (45, 113). While this is the most common adverse manifestation of dual circulation, failing oxygenators may also deliver hypoxemic and or hypercarbic blood distal to the mixing point and contribute to organ dysfunction in that region (18). This scenario is one reason experts advocate for “differential oxygenation” and “differential carbon dioxide” replacing prior nomenclature such as “Harlequin syndrome” or “north-south syndrome” (18). Monitoring for this complication is performed with frequent right upper extremity arterial blood gas sampling, continuous pulse oximetry on the right upper extremity or right ear, use of near-infrared spectroscopy to identify acute cerebral desaturations, and frequent pre- and post- oxygenator blood gas analysis to assess oxygenator function (13). Of note, these monitoring strategies will not detect hypoxemic, hypercarbic blood perfusing the coronary arteries if the mixing point is proximal to the right subclavian artery. Due to the lack of conventional monitoring able to assess coronary oxygenation, the rare situation of isolated coronary hypoxemia should be considered (as a diagnosis of exclusion) if myocardial recovery is not occurring, right upper extremity pulse oximetry and blood gas analysis appears reassuring, but pulmonary pathology exists. One possible method to detect this issue is to very briefly (less than one minute) decrease VA ECMO blood flow rates to low levels (1–2 L/min) with the goal of moving the mixing point distal to the right subclavian artery and drawing an arterial blood gas.

### VA ECMO cannulation for cardiogenic shock

VA ECMO cannulation strategies depend on the urgency of the clinical situation and vascular access considerations. In patients with significant peripheral vascular disease, alternative arterial sites such as the axillary arteries and aorta (via an open approach) may be accessed. For peripheral cannulation, the approach may be surgical (cut down) or percutaneous. One retrospective of 12,592 patients from the ELSO registry of VA ECMO cannulation from 2008 to 2019 in the absence of cardiopulmonary resuscitation favored percutaneous cannulation for multiple outcomes (114). Over the time period studied, 73% of patients were percutaneously cannulated, with an increasing use of this strategy over the time period of the study from 32% to 84% on an annualized basis. Percutaneous cannulation was associated with a reduced risk of infection, bleeding and even mortality while a univariate association with higher severe limb ischemia risk was not replicated in an adjusted analysis, though there was a trend towards an association with more limb ischemia in the percutaneous group (odds ratio 1.28% and 95% confidence interval 0.93, 1.62 in the adjusted analysis) (114). While it is generally recommended to perform percutaneous cannulation, cut down is an option if percutaneous cannulation proves difficult. Additionally, placing the venous cannula on the side contralateral to the arterial cannula (for example, right common femoral venous and left common femoral arterial) is associated with a reduction in the risk of compartment syndrome and fasciotomy, bleeding at the cannulation site, need for vessel repair, and mortality in addition to making decannulation technically more straightforward (108).

Selection of instruments, wires, and techniques used for VA ECMO cannulation is at the discretion of the cannulator. Multiple access needles and dilators are available for percutaneous cannulation including 21- and 18-gauge needles with corresponding 0.018″ and 0.035″ guidewires. While smaller access needles likely contribute to fewer complications, they add additional time to cannulation. Stiff wires have the advantage of making dilation more straightforward, particularly with significant amounts of adipose tissue, but at the cost of being more difficult to advance across acute angled vascular bifurcations (particularly from the left femoral vein). Ultrasound guidance should always be used for percutaneous VA ECMO cannulation as it decreases the time to first vascular access and facilitates access of the common femoral artery instead of accidental puncture of the superficial femoral artery (115–117). Additionally, transthoracic (TTE) or transesophageal echocardiography (TEE) can confirm guidewires in the right atrium (or in the case of TEE, the superior vena cava), identify anatomical abnormalities that could complicate cannulation, and establish a baseline of cardiac function and whether or not an effusion is present prior to cannulation attempts (116–118).

Distal perfusion catheter placement is recommended by the ELSO interim guideline document for VA ECMO, particularly with arterial cannula size above 17 French (13). Pre-emptive distal perfusion catheter placement at the time of cannulation is accepted as a preventative measure for limb ischemia, though even with this intervention, limb ischemia is possible. It is technically straightforward to obtain vascular access for the distal perfusion catheter before placing the arterial cannula. The decision to access the common femoral artery and direct the guidewire towards the superficial femoral artery, or to directly access the superficial femoral artery is at the discretion of the cannulator and is occasionally dictated by patient anatomy and cannulation conditions. A distal perfusion catheter may also be placed in the dorsalis pedis or tibial arteries (13, 102). Distal perfusion catheters are generally 5 to 8 French and are metal reinforced to prevent lumen collapse given the typical acute angle of entry into the vessel (13, 50, 119).

Cannulation may be performed in intubated, sedated patients (most commonly), during the peri-intubation period (obtaining vascular access before the induction of anesthesia and intubation) or in the awake patient (120, 121). The decision to cannulate before or after intubation is made on a case-by-case basis. Patients requiring VA ECMO for right ventricular failure will initially be less tolerant of intubation and may benefit from pre-induction VA ECMO. All patients meeting criteria for VA ECMO initiation in the setting of cardiogenic shock should be considered physiologically difficult airways and managed as such (122–124). If moving forward with cannulation in a non-intubated patient is decided upon, intubation should ideally only be performed when the cannulation team is at the bedside, the field has been prepared, and, unless the patient is completely uncooperative, after initial small bore arterial and venous access is obtained. Cannulation can be completed with surface ultrasound or fluoroscopy in non-sedated patients. Sedation of the non-intubated patient undergoing cannulation should be performed very judiciously to avoid exacerbation of hemodynamic decline and respiratory depression.

### ECMO team composition

The composition of ECMO teams varies by institution, but generally involves a combination of cardiologists, cardiac surgeons, intensivists, perfusionists, respiratory therapists, nurses, physician assistants, nurse practitioners, pharmacists and many other healthcare professionals. ECMO teams often delegate a group of clinicians to decide upon initiation when a VA ECMO consult for cardiogenic shock is placed. Decision teams should not be large as this can delay timely initiation. However, a single clinician should not be the sole decision-maker as this approach fails to sufficiently account for all aspects of the clinical situation and can lead to both missed opportunities and individual risk due to a lack of shared responsibility. While certain situations are relatively straightforward, decision teams should be employed in most cases. Literature supports the implementation of collaborative ECMO teams and demonstrates associations with significant improvements in outcomes including mortality (125–127).

VA ECMO cannulation may be performed by clinicians with demonstrated technical skill; fields such as intensive care, interventional cardiology, cardiac surgery, and emergency medicine lend well to the desired skill set (128, 129). Importantly, for any given institution, the pool of cannulators should be kept relatively small to avoid atrophy of skills and variability. While a specific number of cannulations is not known, it is unlikely that skills can be maintained in a setting in which an individual participates in less than 10 cannulations per year. Very low volume centers (less than 10 to 20 cannulations per year) may be best suited with one to two cannulators and establishment of a relationship with a higher volume institution.

Daily management is best carried out in multidisciplinary fashion by intensivists with cardiovascular experience, heart failure cardiologists, and cardiac surgeons (130). For patients considered for advanced heart failure therapies, the corresponding heart failure team should be heavily involved in care as management decisions can impact candidacy and have procedural implications. Protocols for the monitoring of limb ischemia, hemolysis, neurologic sequelae, and weaning should all be in place to minimize and immediately address complications. Communication is key and bedside clinicians should feel empowered to raise concerns. Quality improvement is an integral part of any ECMO program, and any clinician involved must submit to attending meetings regarding ECMO-related complications for program growth.

### VA ECMO circuit parameters

VA ECMO circuit parameters include sweep gas flow, fraction of delivered oxygen (F_d_O_2_), the blood flow rate (modified via revolutions per minute or RPMs), and thermal regulation. The sweep gas flow rate is the rate of gas entering the oxygenator (generally 1 to 10 L/min), which when increased, will clear more carbon dioxide and when decreased clears less carbon dioxide from the blood entering the oxygenator. The F_d_O_2_ is the fraction of oxygen being delivered to the oxygenator and can be varied using a blender from 0.21 to 1.0 (21% to 100%). A registry-embedded randomized trial of 300 patients found no difference in the primary outcome (ICU free days from day 28) or mortality at various time points in VA ECMO patients assigned to a target patient oxygen saturation of 92% to 96% vs. 97% to 100% (via both post oxygenator and right upper extremity arterial blood gases) (131). The VA ECMO circuit blood flow rate is manipulated by setting motor RPMs and is measured by non-invasive flow probes attached to the ECMO circuit tubing. Positive displacement roller pumps will generate blood flow based solely on stroke volume, but more commonly used centrifugal pumps are afterload sensitive so RPMs must be adjusted to maintain target blood flow. Higher blood flows will provide more hemodynamic support but increase the risk of hemolysis. The ideal target blood flow in VA ECMO is not currently known and likely depends on clinical circumstance. One ELSO registry analysis presented as an abstract found that in the absence of mechanical LV unloading, a VA ECMO flow index below 2 L/min/m^2^ is associated with lower mortality than 2 L/min/m^2^ or more (132). The heater-cooler assists with thermoregulation and can be used to reduce body temperature, reducing metabolic demand and increasing systemic vascular resistance. The need to reduce warming bath temperature to maintain normothermia may be indicative of fever due to infection or other causes. Without these units, patients are less likely to consistently remain normothermic (133).

Monitored parameters for VA ECMO patients include pre and post oxygenator blood gas analysis (which determines oxygenation and ventilation by the membrane lung), pre- and post- oxygenator pressures, and regular direct assessment of the circuit for thrombus and fibrin deposition. Pre and post oxygenator blood gases should demonstrate a step up in oxygen partial pressure and a reduction in carbon dioxide partial pressure to deliver adequately oxygenated and ventilated blood distal to the mixing point. Failure of either oxygenation or ventilation is an indication for oxygenator exchange, and they need not fail simultaneously (134). Institutional protocols generally determine when oxygenator exchange is necessary. This is a high-risk period given the need to briefly stop circuit flows and because, following exchange, the same level of sweep gas flow may result in more efficient clearance of carbon dioxide and thus risk neurologic complications, such as seizures or intracranial hemorrhage, stemming from acute large swings in P_a_CO_2_.

### VA ECMO management

Daily management of the VA ECMO patient is carried out by a multidisciplinary team. All organ systems must be meticulously reviewed in the discussion of management with additional considerations given for ECMO circuit function. Monitoring and management of the patient supported by VA ECMO includes evaluating neurologic function for acute brain injury; assessing for adrenal insufficiency and glycemic control; right upper extremity blood gas monitoring (in cannulation strategies other than central or right axillary arterial cannulation) for adequate cerebral oxygenation; mechanical ventilator management; vigilance for acute liver injury and the effects on synthetic function; renal function including fluid balance; electrolyte monitoring and acid-base balance, limb perfusion; and hematological considerations. Infections are common in the VA ECMO population and are of varying etiologies with incidence difficult to quantify (89). Cardiovascular monitoring considerations include the assessment of adequate perfusion, discerning vasoplegia from insufficient total blood flow, maintenance of pulmonary and systemic arterial pulsatility, planning of further interventions and de-escalation of VA ECMO support, volume removal, anticipation of fluid shifts, interpretation of hemodynamics and decision-making surround mechanical unloading. Pulmonary artery catheterization, arterial access, and echocardiography form the cornerstones of hemodynamic monitoring in this population and must all be reported with vasoactive agent dosing and support levels including blood flow rates of all tMCS devices. VA ECMO circuit parameters (fraction of delivered oxygen, sweep gas flow rate, revolutions per minute) must be reviewed and titrated to maintain homeostasis and adequate perfusion with a focus on an adequate ratio of oxygen delivery to consumption (greater than 3) (13). Additionally, the status of the VA ECMO circuit (including pre and post oxygenator blood gases, the delta pressure across the oxygenator, surveillance of clot accumulation within the tubing and oxygenator) must be closely monitored to anticipate circuit failure. Pharmacologic considerations in the VA ECMO population are complex given alterations in volume of distribution and drug sequestration.

The management and prevention of neurologic issues in the VA ECMO population is a major challenge given the high incidence of intracranial pathology in this population. Neurologic monitoring is accomplished with spontaneous awakening trials, cerebral oxygen saturation monitoring via near-infrared spectroscopy, brain injury biomarkers (including neuron specific enolase), pupillometry, processed electroencephalogram monitoring, and computed tomography (CT) scans (76). Physiologic targets are a subject of ongoing investigation, with a recent ELSO neuromonitoring guideline document acknowledging a lack of evidence and recommending avoidance of severe patient hyperoxemia (as defined by a partial arterial pressure of oxygen, P_a_O_2_, above 300 mmHg), maintaining a P_a_O_2_ of 70 mmHg or greater, a mean arterial pressure (MAP) above 70 mmHg, avoidance of acute swings in P_a_CO_2_, and maintenance of normothermia (preventing temperatures above 37.7 C) (76). The authors also state that induced hypothermia (to 33–36 C) may be reasonable (76, 135). For VA ECMO, induced hypothermia may be favorable because, unlike in patients not supported by VA ECMO, hypothermia should not affect circuit flows, whereas in patient not supported by VA ECMO, cardiac output will decrease with hypothermia and thus the ratio of oxygen delivery to consumption may be more favorable. In patients with suspected ischemic stroke, immediate neurological consultation and non-contrast head CT are recommended in addition to avoiding tissue plasminogen activator (tPA) in favor of thrombectomy with neurointerventional radiology (76). For the case of ischemic stroke, and for intracranial bleeding, the authors suggest stopping anticoagulation with the acknowledgement that the risk of VA ECMO circuit related complications is somewhat elevated but acceptable (76).

Pulmonary management of patients supported by VA ECMO requires an understanding of the dual circulation present in non-right axillary peripheral VA ECMO. Mechanical ventilator management should involve low tidal volume ventilation with PEEP and judicious sweep titration given the association between rapid P_a_CO_2_ changes and neurologic complications. Among other findings, a survey of mechanical ventilation in the ECMO population found that the most commonly applied PEEP is 10 cmH_2_O or less, the most frequently used sedatives are propofol, midazolam and dexmedetomidine, and that driving pressures of below 15 cmH_2_O were targeted (136). In this same survey, most centers (62%) targeted tidal volumes below 6 mL/kg but 23% targeted greater than 6 mL/kg (136). Data on the appropriate mechanical ventilator settings in this population is sparse and should be guided by clinical intuition and the physiologic targets needed by the patient while maintaining lung protection.

Cardiovascular considerations in the VA ECMO patient include monitoring perfusion markers, stabilizing hemodynamics, determining total effective blood flow, and decision-making surrounding LV mechanical unloading. Continuous, real-time monitoring with right upper extremity arterial access and pulmonary artery catheterization are necessary to monitor both oxygenation and ventilation, titrate vasoactive medications, determine fluid balance goals, and trigger LV mechanical unloading. Pulmonary artery catheter use has been associated with improved mortality in patients with cardiogenic shock in multiple large retrospective reviews, and is recommended for VA ECMO by the ELSO interim VA ECMO guideline document (13, 137, 138). Pulmonary artery catheters can assist with immediate identification of the etiology of low pulse pressure (low filling pressures would suggest the need for volume administration while high filling pressures may suggest LV failure), are important for weaning VA ECMO, and assist with diuresis. Interpretation of pulmonary artery catheter-derived hemodynamics in the VA ECMO population is challenging due to the drainage of blood from the venous system (139). The determination of systemic vascular resistance requires an understanding of total body flow in the VA ECMO population. For example, if a patient is supported by 4 L/min of VA ECMO circuit flow and has a LV outflow tract velocity time integral suggestive of 2 L/min of native cardiac output, then total flow is 6 L/min assuming minimal or no aortic insufficiency. In this example, if the patient has a body surface area of 2.5 m^2^, then indexed flow is 2.4 L/min/m^2^ and hypotension would likely be due to low systemic vascular resistance and should be treated accordingly (140).

When LVAD or heart transplantation from VA ECMO is planned, additional considerations exist. For heart transplant candidates, judicious use of transfusion is necessary to avoid antibody generation, which can complicate finding a suitable donor, jeopardizing the opportunity for transplant. Additionally, extubation should especially be considered when feasible in this patient population as there may be an association with improved outcomes (120, 141). For patients being bridged to LVAD, avoidance of atrial septostomy and measures to prevent renal dysfunction are crucial. Atrial septal defects must be repaired at the time or prior to LVAD placement due to the potential for right to left shunt upon device activation. Additionally, patients with significant renal dysfunction or the need for dialysis tend to have poor outcomes when undergoing durable LVAD implantation. The use of VA ECMO as a bridge to durable LVAD and heart transplant is increasing and centers that do not perform these procedures should immediately transfer these patients (once stabilized) to centers offering these therapies so that they may receive specialized care.

LV mechanical unloading is extensively discussed in the literature. Hemodynamic thresholds for this intervention are not known, and clinicians must be cognizant of the associated complications including bleeding, thrombocytopenia, hemolysis, limb ischemia and acute brain injury (142, 143). For most patients supported by VA ECMO with or without IABP, increasing circuit flows results in a reduction in PCWP due to reduced preload, with only 6% of patients developing an increase in one study (44). This suggests that the number of patients requiring LV mechanical unloading is likely lower than previously thought and may explain why trials examining indiscriminate unloading have thus far been negative (144, 145). Options for LV mechanical unloading include IABP, tvMFP, left atrial septostomy, left atrial cannulation, direct LV venting using a surgically placed cannula, catheters traversing the aortic valve to drain blood from the LV, and drainage of the pulmonary artery (Table 5) (13, 43). In general, LV mechanical unloading is indicated if significant pulmonary edema exists that cannot be addressed less invasively, if systemic arterial pulsatility is 10 mmHg or less despite inotropes and volume optimization, or if arrhythmias occur that are thought due to cardiac distention. Hemodynamic triggers are not known and have not been studied. While it seems reasonable to perform LV mechanical unloading in patients with a PCWP of 18 to 20 mmHg, less invasive maneuvers should be attempted first (13). A large ELSO registry study of mechanical unloading from 2010 to 2019 found that of 12,734 patients, 3,399 (26.7%) were unloaded and that of these, 2,782 (82.9%) received IABP and 580 (17.1%) received tvMFP (143). Mortality was lower in the patients undergoing unloading (56.6% vs. 59.3%), but the risk of cannula site bleeding and hemolysis were higher (143). Additionally, there was no mortality difference between IABP and tvMFP, but a higher rate of medical bleeding, cannula site bleeding, and hemolysis occurred in the tvMFP group (143). Unfortunately, this work did not include hemodynamics and thus cannot answer the question of thresholds to pursue mechanical unloading in patients that do not have clinical indications. Ultimately, the decision to unload a patient should be based on a well-reasoned argument and should not be done without first analyzing hemodynamics given the risk of significant complications.

**Table 5:** Selected mechanical LV unloading options with advantages and disadvantages (43, 50, 143).

Mechanical Unloading Strategy	Advantages	Disadvantages
IABP	Smaller arteriotomy (9 Fr) Relatively easy to deploy (possible at bedside) High dose AC not needed	Requires some cardiac activity Worsens aortic insufficiency High risk in PVD Contraindicated in dissection, significant AI
tvMFP	Higher blood flow Does not require residual cardiac activity	Requires transport to procedure suite Larger arteriotomy (14 Fr) High risk in PVD Contraindicated in LV thrombus, ASD, VSD, 2+or worse AI, AS with valve area less than 0.6 cm^2^
Transseptal cannula (LA drainage)	No arterial Access (venous access with transseptal puncture) No contraindication in LV thrombus Requires anticoagulation due to persistent left atrial access Potentially better option in PVD	Requires OR trip, Risks of transseptal puncture (pericardial effusion, access complications), need to maintain reliable blood flow to prevent thrombosis, RV dysfunction will reduce blood flow to the left atrium and decrease drainage
Atrial Septostomy	No arterial Access (Venous access plus transseptal puncture) No contraindication in LV thrombus Potentially better option in PVD	Requires transport to procedure suite Risks associated with transseptal puncture (pericardial effusion, access complications), Variable unloading
Percutaneous LV catheter drainage (trans-aortic valve)	Access via radial artery Independent of LV function	Caution in LV thrombus Limited drainage capability Limited experience
Surgical LV Drain	High blood flow rate, Direct Unloading, no LV function needed	Surgical approach (OR trip) Thrombosis (need to maintain flow through drainage cannula), bleeding

AC, Anticoagulation; AI/AS, aortic insufficiency/stenosis; ASD, Atrial septal defect; cm, centimeters; IABP, Intra-aortic balloon pump; LA, left atrium; LV, left ventricle; OR, operating room; PVD, peripheral vascular disease; RV, right ventricle; tvMFP, transvalvular microaxial flow pump; VSD, ventricular septal defect.

LV mechanical unloading is most commonly performed with IABP or tvMFP. IABP provides left ventricular (LV) support and increased coronary perfusion by inflating during diastole (T wave) and deflating during systole (on the R wave) (146). The IABP can also trigger by pressure with inflation at the dicrotic notch and deflation just before systolic upstroke (146). Although it is most commonly deployed through a common femoral artery, the IABP may also be placed via axillary or subclavian arteries, or directly via the aorta. The IABP requires some residual LV activity to unload, and the artificial pulsatility it generates could mislead clinicians that the aortic valve is opening when it is not (147). In addition to LV unloading during VA ECMO, the IABP is used frequently in the setting of cardiogenic shock, as well as in severe, symptomatic coronary artery disease (43). The tvMFP is a continuous flow axial flow pump that traverses the aortic valve and propels blood from the LV to the aorta (43, 148). Multiple sizes exist, but the smallest is deployed via a 14 French catheter, typically in the common femoral artery. Advantages of tvMFP over IABP include more powerful unloading (nearly 6 L/min for the largest model), and that residual myocardial activity is not necessary for it to mechanically unload the LV while disadvantages are the larger sheath size, and higher complication rate (43, 143).

Hematologic considerations in the VA ECMO population include anticoagulation and transfusion thresholds. Ideal transfusion thresholds in this population are not known, with one recent work suggesting a potential early (first three days of VA ECMO) benefit with the use of a hemoglobin threshold of 9 g/dL or more compared to 7 g/dL (149, 150). Patient factors must be considered when transfusion is being considered. For example, patients being considered for heart transplant may benefit from a lower hemoglobin (7 g/dL) transfusion threshold to minimize antibody formation from multiple blood transfusions, while patients supported for recoverable indications may benefit from higher transfusions thresholds. Transfusion thresholds for platelets and other blood products are not known and are dictated by patient condition and bleeding. Anticoagulation is minimally studied in the VA ECMO population, but an ELSO guideline document recommends the use of unfractionated heparin with a goal anti-xa level of 0.3 to 0.7 units/mL, though high bilirubin and the use of hydroxocobalamin can artificially alter the results of this assay (151, 152). Of note, evidence exists supporting the use of bivalirudin and other direct thrombin inhibitors for anticoagulation in this patient population (153). While anticoagulation is highly recommended, one retrospective review suggests that VA ECMO without anticoagulation may be safe, though patients with significant blood stasis are likely at high risk of thrombus formation with this strategy (154).

### Pharmacologic alterations on ECMO

Medical management of patients supported by ECMO requires careful consideration of drug selection, dosing, and adjustment. As blood is exposed to a very large surface area of circuitry components, ECMO introduces complexity to traditional drug pharmacokinetic models and has serious management implications. Unlike dialysis or apheresis, which produce a substance-rich effluent, ECMO is a closed system. Because of this, drugs do not follow a traditional elimination mechanism as they would with native organ clearance or extracorporeal removal like dialysis or apheresis. As such, quantification of drug alteration, and subsequently decision-making and management is challenging in ECMO.

The physiochemical properties of drugs influence their propensity for altered behavior during ECMO support. High-quality evidence regarding drug disposition on ECMO is lacking; however, knowledge is extrapolated from ex-vivo ECMO models, animal experiments, and human studies (155). Patient populations represented among studies is highly variable and includes neonates, children, and adults (155). With ECMO, drugs with high lipophilicity, low volume of distribution, and high protein binding tend to have the most deranged pharmacokinetics (156).

Upon ECMO initiation the circuit increases the total effective circulatory volume. These effects are more pronounced in smaller patients and account for approximately 20 to 30% of circulating volume (157). These hemodilution effects have important implications on drug volume of distribution, and in some situations may warrant increased doses or additional bolus doses depending on the extent of alteration and how narrow the therapeutic window for the drug is (158). In addition to prime volume, type of fluid used for circuit prime (e.g., crystalloid, colloid, blood, or a combination) must be considered as this may also impact expected drug kinetics (159).

During ECMO support, the large surface area of plastic tubing used to connect circuit components, the centrifugal pump, and membrane oxygenator present numerous opportunities for drug binding (so-called “sequestration”) (160). Older-generation membrane oxygenators were composed of polypropylene fibers. Their hydrophobic surfaces allowed for gas exchange to occur across micropores without blood leakage to the fiber compartment and vice versa (161, 162). However, these pores provide a prime medium for drug deposition, particularly large lipid-based substances, which were known to quickly exhaust the devices (163). Newer generation membrane oxygenators are composed of a dense network of polymethylpentene fibers. While these fibers are non-porous or nanoporous allowing for gas exchange to occur across a diffusion gradient, experimental studies and case reports suggest that cellular material and drugs may still deposit (164–167).

Circuit configuration and support settings may also have potential consequences on drug disposition. Patients supported by VA ECMO have a large portion of blood flow diverted from the native pulmonary circulation, and depending on the scenario, this may be upwards of 75 to 90% of total blood flow. Theoretically, this poses a risk for suboptimal drug delivery to pulmonary tissue, which may have implications for treating infections. No evidence exists to corroborate or dispute this phenomenon, but vigilance is warranted. Additionally, recent evidence suggests that high ECMO RPM or blood flow may accelerate drug clearance. A Bayesian population pharmacokinetic study developed a model utilizing RPM and gender that effectively predicted remifentanil clearance (168). At fixed infusion rates, remifentanil clearance increased with increasing RPM, an effect that was more pronounced at higher infusion rates. Given remifentanil's esterase metabolism, plasma turnover may be a potential explanation for this; however, another study found similar effects of RPM and ECMO flow rate on midazolam clearance, which might be attributed to greater hepatic blood flow (169).

Lastly, while the physiochemical properties of drugs and technical characteristics of the ECMO circuit are important, they are not the only factors that should be used to guide drug dosing decisions. Because of their underlying cardiopulmonary disease, most patients supported by ECMO suffer from critical illness and the accompanying physiological disturbances that also contribute to drug disposition such as acid-base disorders, capillary leak syndrome, altered protein binding, and end-organ dysfunction (156). Furthermore, the presence of other devices, such as dialysis, apheresis, and blood filters, that may contribute to drug clearance must be considered. Great caution is warranted when extrapolating evidence from experimental studies, such as ex-vivo models, to the bedside (155, 170).

### VA ECMO weaning and decannulation

VA ECMO decannulation is indicated when a patient's cardiopulmonary status tolerates a “turn down” or “pump controlled retrograde trial off” (RPM reduction to allow retrograde flow from arterial to venous systems with flushing and clamping of the distal perfusion catheter) (13, 171, 172). Institutional practice varies widely. Multiple decision-making cutoffs exist including adequate cardiac index (2.2 L/min/m^2^) with central venous pressure (CVP) at most 12 mmHg at minimal VA ECMO circuit flow (1 L/min maximum) for at least 10 min, and PCWP at most 15 mmHg with one inotrope and or one vasopressor at moderate doses (13). During weans, other support devices such as IABP or tvMFP are typically continued or support escalated. For patients supported by VA ECMO for cardiogenic shock, weaning should only occur when the etiology of cardiogenic shock has been addressed, which may entail revascularization of coronary artery disease, volume optimization, immunosuppression for myocarditis, and resolution of stress cardiomyopathy. Volume optimization should be accomplished by diuresis or renal replacement therapy if indicated, and myocardial recovery should be noted by invasive hemodynamics and echocardiographic evaluation with minimal doses of vasoactive agents. For example, guidelines and literature recommend the patient should have a negative fluid balance in the days before weaning, and arterial blood gas analysis should demonstrate adequate oxygenation and ventilation with a ventilator inspired oxygen level at most 60% and plateau and driving pressures consistent with lung protective ventilation (13, 171). One strategy for weaning is as follows: when cardiorespiratory function is adequate on partial blood flow, for example, a blood flow of 2 L/min for at least several hours, institutional protocols to increase anticoagulation should be followed and VA ECMO blood flow can be reduced to 1 L/min for at 10 to 15 min, hemodynamics and respiratory function assessed, and after blood flows are restored to prevent thrombotic complications, a joint decision on decannulation may be performed when anticoagulation status is improved. In tenuous patients, cannulas may be flushed with saline and the circuit connected to itself and running so that rapid re-institution can occur in the event of decompensation.

Specific guidance on the weaning of VA ECMO is also found in a scientific statement from the American Heart Association by Geller et al. (173) A daily evaluation for weaning suitability is recommended upon hemodynamic stabilization, and respiratory status should be acceptable, with Geller et al. recommending a P_a_O_2_ to fraction of inspired oxygen ratio of at least 200 (173). Importantly, respiratory status should be stable without lung injurious ventilator settings, or labored breathing patterns if not intubated. After adequate anticoagulation, the authors recommend reducing blood flow by 0.5 to 1 L/min at a time (until 1–2 L/min has been reached) in two possible ways: an expedited approach which constitutes weaning by this flow rate every 5 to 15 min, and a more gradual option of decreasing flows every 2 to 4 h (173). Monitoring with pulmonary artery catheterization, echocardiography, and markers of perfusion to guide the weaning process is also suggested (173). In particular, the authors recommend a CVP below 10 to 15 mmHg and PCWP below 18 mmHg while maintaining a MAP of at least 65 mmhg and lactate at most 2 mmol/L using minimal vasoactive agent support when minimum blood flows are reached (173). Finally, the authors appropriately state that weaning should be individualized as certain patients (such as those with chronic heart failure) may be weaned with differing hemodynamics and vasoactive support than typical, and that those with complications related to tMCS may require weaning before meeting usual criteria to avoid further complications (173).

Decannulation of the arterial limb most commonly proceeds in one of two ways: surgical (cut down and vessel repair) or with percutaneous closure devices. Debate exists on the optimal decannulation approach with meta-analyses suggesting a potential improvement in certain outcomes with percutaneous closure (174, 175). It should be noted that significant peripheral vascular disease is a contraindication to percutaneous closure and thus surgical closure may be needed in that patient population. Additionally, the surgical approach is the bailout when percutaneous attempts fail, and thus surgical availability should be present when percutaneous decannulation attempts are made. The distal perfusion catheter is generally removed with manual pressure, though that arteriotomy may be surgical repaired as well. Venous cannula removal is straightforward and generally is accomplished with manual pressure.

### Post VA ECMO decannulation care

After VA ECMO decannulation, mortality remains high with 24-hour post decannulation survival of 62.6%, and 44.2% survival at hospital discharge (an 18.2% absolute survival reduction) (91). After decannulation, a systemic inflammatory response can occur manifesting as vasodilation, though infection is also possible (176, 177). Additional causes of morbidity and mortality after decannulation include bleeding from decannulation sites, limb ischemia from embolism after arterial cannula removal, pulmonary embolism (given the high rate of cannula-associated deep vein thrombosis rate), recurrence of cardiogenic shock, pseudoaneurysm, multi organ dysfunction, and infection. One study of 109 patients after VA ECMO decannulation found that computed tomography evidence of cannula associated venous thrombosis was 50%, and arterial thrombosis was also common at 33%, and bleeding was noted in 31% of patients (178). After VA ECMO decannulation, organ function should be evaluated frequently and it is necessary to assess multiple markers including invasive hemodynamics, acid-base status, blood lactate levels, urine output, creatinine, and liver function tests. Changes in these markers should prompt immediate hemodynamic assessment, volume optimization, and decisions regarding re-institution of tMCS. Importantly, due to the high incidence of post-decannulation bleeding and systemic inflammatory response, the etiology of cardiovascular decline must be rapidly discovered (for example, immediate assessment of decannulation sites) to perform appropriate care (177, 178).

In general, maintaining other tMCS (for example, IABP or tvMFP) and inotropic support (unless complications arise) for a short period after decannulation allows for stepwise de-escalation. Similarly, immediate extubation could result in myocardial stress and should be avoided in the first 12 to 24 h after VA ECMO decannulation. If recannulation is necessary, accessing the vessels not previously accessed, or, in the case of surgical repair, reopening the wound and cannulating under direct visualization are options. Although survival is lower after multiple ECMO runs, recannulation should not necessarily be considered futile (179, 180).

## Discussion

VA ECMO for cardiogenic shock is a highly resource intensive support strategy that has significantly affected care of this population. Despite increasing use of VA ECMO, many unanswered questions remain, including definitive evidence of outcome benefits of this highly invasive support modality, timing and criteria for cannulation and decannulation, changing from VA ECMO to other tMCS strategies, anticoagulation goals, flow and physiologic targets, and appropriate cannula sizing. Complications in patients supported by VA ECMO for cardiogenic shock are common and include hemocompatibility issues (such as bleeding, thrombosis, and hemolysis), neurologic (including stroke, intracranial hemorrhage and hypoxic brain injury), pulmonary complications such as pulmonary edema, acute kidney and liver injury, limb ischemia, and physiologic consequences such as differential oxygenation. Multidisciplinary care is necessary in this complex patient population. Additionally, there is a paucity of data on long-term outcomes and quality of life after VA ECMO support because large databases often do not record outcomes after hospital discharge. VA ECMO is a promising technology, and important questions must be answered to improve patient outcomes.
